# Few gender differences in specialty preferences and motivational factors: a cross-sectional Swedish study on last-year medical students

**DOI:** 10.1186/1472-6920-13-39

**Published:** 2013-03-08

**Authors:** Saima Diderichsen, Eva E Johansson, Petra Verdonk, Toine Lagro-Janssen, Katarina Hamberg

**Affiliations:** 1 Department of Public Health and Clinical Medicine, Family Medicine, Umeå University, Umeå, 901 85, Sweden; 2 Department of Medical Humanities, VU Medical Center, EMGO Institute for Health and Care Research, Amsterdam, The Netherlands; 3 Department of General Practice and Community Care, Institute for Gender Studies, Radboud University Nijmegen Medical Centre, Nijmegen, The Netherlands

## Abstract

**Background:**

Today, women constitute about half of medical students in several Western societies, yet women physicians are still underrepresented in surgical specialties and clustered in other branches of medicine. Gender segregation in specialty preference has been found already in medical school. It is important to study the career preferences of our future physicians, as they will influence the maintenance of an adequate supply of physicians in all specialties and the future provision of health care. American and British studies dominate the area of gender and medical careers whereas Swedish studies on medical students’ reasons for specialty preference are scarce. The aim of this study is to investigate and compare Swedish male and female medical students’ specialty preferences and the motives behind them.

**Methods:**

Between 2006 and 2009, all last-year medical students at Umea University, Sweden (N = 421), were invited to answer a questionnaire about their future career and family plans. They were asked about their specialty preference and how they rated the impact that the motivational factors had for their choice. The response rate was 89% (N = 372); 58% were women (N = 215) and 42% were men (N = 157). Logistic regression was used to evaluate the independent impact of each motivational factor for specialty preference.

**Results:**

On the whole, male and female last-year students opted for similar specialties. Men and women had an almost identical ranking order of the motivational factors. When analyzed separately, male and female students showed both similarities and differences in the motivational factors that were associated with their specialty preference. A majority of the women and a good third of the men intended to work part-time. The motivational factor combining work with family correlated with number of working hours for women, but not for men.

**Conclusions:**

The gender similarities in the medical students’ specialty preferences are striking and contrast with research from other Western countries where male and female students show more differences in career aspirations. These similarities should be seized by the health care system in order to counteract the horizontal gender segregation in the physician workforce of today.

## Background

In the U.S. and in Europe, the number of women in medical schools started to increase in the 1970s [1,2]. The careers of these female physicians were expected to follow a linear development in all fields and levels of medicine. In the late 1990s, the time-lag phenomenon no longer could explain women’s lack of career advancement in male-dominated specialties such as surgery, and research on gender and medical careers started to grow [2]. Today, when women constitute about half of medical students in several Western societies [3-6], statistics still show that women physicians are underrepresented in high-status specialties like surgery and clustered in specialties characterized by relatively low earnings or prestige [7-9]. Gender segregation in specialty preference has been found already in medical school [10-12] and previous studies have not been able to show any changes in this remarkably stable and almost universal pattern [1,13,14].

The motives behind specialty preference have been studied, both on an individual level and on a structural level. On an individual level it has been revealed that female students consider work- and time-related aspects and patient orientation whereas their male peers consider technical challenge, salary, career prospects and prestige [11,15-18]. It has also been shown that medical students and young physicians of both genders value lifestyle factors and consider balance between work and private life [19-22]. On a more structural level, lack of social support and a scarcity of role models have been shown to affect men and women’s specialty preference [1,16,23]. Moreover, the scarcity of female physicians in surgery has been found to be a result of a masculine exclusionary practice and a consequence of women’s choices being made in relation to their immediate or expected family duties [24].

That women are expected to and generally do take more responsibility for domestic work has been a common explanation for gender differences in men’s and women’s individual career choices. There is a national context that needs to be taken into consideration here, as career choices are always made within a frame of reference, composed of the possibilities and limitations that can be found in a certain context [25]. American and British studies dominate the area of gender and medical careers [2]. However, their social policies differ from that in Sweden, where paid parental leave and subsidized childcare encourage women and men to share paid and unpaid work equally [26]. Social policies of this type affect individuals’ behavior: how they choose their careers and organize their private lives [25]. Therefore, Sweden is a good place to study male and female medical students’ career preferences, as the norms of gender equality are relatively strong, especially among the highly educated [26,27]. To our knowledge, Swedish studies on medical students’ reasons for specialty preference are scarce. Medical students’ career preferences per se are important to study, as their choices will influence the maintenance of an adequate physician supply and the future provision of health care. Therefore, the objective of our study is to compare specialty preferences and motivational factors of male and female graduating medical students. We will investigate associations between motivational factors and specialty preference in order to determine reasons contributing to specialty choices.

## Method

### Study population

This cross-sectional study is part of the project Gender Challenges in Medical Education [12]. Between 2006 and 2009, all classes of last-year medical students at Umea University in Sweden (N = 421) were asked to complete a confidential questionnaire (58% women, 42% men). The students could stay on after an ordinary lecture and fill out the survey. The response rate was 89% (N = 372). Among the respondents, 58% (N = 215) were women and 42% (N = 157) were men.

### The questionnaire

The questionnaire included socio-demographic questions such as students’ sex, age and family status. The students were asked to choose one out of seven specialties or the options *something else* and *I don’t know*. Under *something else* the students added eight more. In order to create larger categories these 15 specialties were merged into three groups: Surgical specialties (general surgery, otolaryngology, ophthalmology, anesthesiology and gynecology), non-surgical specialties (internal medicine, pediatrics, psychiatry, neurology, venereology, infectious diseases, radiology, oncology and laboratory specialties) and family medicine. Surgical specialties and family medicine were separated as they differ both in content and in number of hours on call. The non-surgical group was relatively diverse as we merged specialties such as pediatrics, internal medicine and laboratory medicine into the same group. Students who opted for two or more specialties from different specialty groups were merged with students who chose “I don’t know”. This group was named “uncertain”. There was no distinct pattern in the group that opted for two or more specialties. The combinations ranged from two to six different specialties and there was no combination that occurred more than twice.

Based on literature, ten motivational factors that might contribute to the students’ preference for specialties were defined [28,29]. The students were asked to rate (Likert scale 1 to 5) how important these factors were when they were to choose a specialty. Three of the motivational factors were deleted from the analysis. The two factors: *in line with my former student experience* and *in line with my former work experience* were taken away since they had no association with the specialty preferences. The third factor, *attractive working hours,* was excluded, as it correlated strongly with the factor *combining work with family* in both women and men. There was also a specific question where the students were asked to state ideal working hours, and that question was used in a separate analysis.

### Statistical analyses

Binary logistic regression was applied to investigate the associations between motivational factors and specialty preference. In this regression analysis men and women were analyzed separately. To assess gender differences in these links between motivational factors and specialty preference, gender was combined with each motivational factor as interaction term. Also, a linear regression analysis was conducted in order to reveal any correlation between preferred number of working hours and the rating of the motivational factor *combining work with family*.

Analysis was made in SPSS 20.0 for Mac and the Pearson chi-square test was used to determine the significance of specialty preferences and motivational factors. To reveal any significant gender differences in the rating of the motivational factor we used the non-parametric Mann–Whitney *U* test. The significance was set at p < .05.

The Regional Ethical Review Board at Umea University has approved this study.

## Results

### Socio-demographics

About half of both male and female students lived together with a partner (see Table 1). A higher proportion of men than women had children, but this was not a significant difference. A vast majority of the students had highly educated (university education) parents who worked full-time (40 hours or more per week). More mothers than fathers worked part-time, reflecting gender differences in the general population. Many of the students’ parents were retired, which partly explains the high proportion in the variable *other* under *working status father* and *working status mother*.

**Table 1:** Socio-demographics of participating male and female students

**Category**	**Variable**	**Women**		**Men**		**p**
		**(N = 216)**		**(N = 157)**		
		**Mean (SD)**		**Mean (SD)**		
Age		27.3	(3.2)	27.7	(3.2)	NS
		**%**	**(N)**	**%**	**(N)**	
Civil status	Not cohabiting	50	(107)	46	(73)	NS
	Cohabiting	50	(109)	54	(84)	
Children	No	89	(192)	83	(131)	NS
	Yes	11	(24)	17	(26)	
Highest education mother	Higher	73	(157)	78	(121)	NS
	Intermediate	20	(43)	17	(27)	
	Primary	7	(14)	5	(7)	
Highest education father	Higher	64	(137)	75	(116)	**.042**
	Intermediate	22	(47)	18	(27)	
	Primary	14	(30)	7	(11)	
Working status mother	Full-time	60	(129)	71	(112)	NS
	Part-time	18	(38)	14	(22)	
	Other	22	(48)	15	(23)	
Working status father	Full-time	69	(148)	68	(106)	NS
	Part-time	5	(10)	6	(10)	
	Other	27	(58)	26	(41)	

*Note:* Significance level for gender differences was set at p < .05. SD = Standard deviation. NS = not significant. p < .05 in bold.

### Gender and career preferences

The most frequently preferred specialties among both men and women were surgery, family medicine and internal medicine (Table 2). Gynecology and pediatrics were also rather common, especially among women. Almost a third of the students were uncertain of their specialty preference. This might seem high, but graduated medical students in Sweden still have at least one and a half years of internship before they can go into residency training. A total of four students (three men and one woman) were not included in this analysis, as they had chosen “other” without specifying which other specialty. There was only one statistically significant gender difference; more women than men chose gynecology.

**Table 2:** Male and female students’ career preferences

**Variable**	**Category**	**Women**		**Men**		**p**
		**N = 215**		**N = 157**		
		**%**	**(N)**	**%**	**(N)**	
Specialty preference	**Surgical specialties**	27	(58)	29	(44)	NS
	Surgery	17	(36)	23	(36)	NS
	Gynecology	8	(17)	3	(4)	**.028**
	Anesthesiology	2	(5)	3	(4)	NS
	**Non-surgical specialties**	22	(48)	24	(37)	NS
	Internal medicine	8	(17)	9	(14)	NS
	Pediatrics	7	(15)	4	(6)	NS
	Psychiatry^a^	3	(6)	3	(5)	NS
	Neurology	2	(4)	3	(5)	NS
	Others	3	(6)	5	(7)	NS
	**Family medicine**	18	(38)	17	(26)	NS
	**Not sure**	32	(68)	30	(47)	NS
Preferred working hours (hours per week)	More than full-time (>40)	2	(5)	10	(15)	**.001**
	Full-time (40)	40	(85)	51	(80)	
	Slightly less than full-time (31–39)	23	(50)	12	(19)	
	75% and less (≤30)	31	(66)	24	(37)	
	Other	4	(9)	4	(6)	

*Note:* Significance level for gender differences was set at p < .05. NS = not significant. p < .05 in bold. ^a^Psychiatry includes child and adolescent psychiatry.

A large proportion of both men and women preferred part-time practice (see Table 2). It was however significantly more common among the women; a majority of the female students (54%) compared with a good third their male peers (36%) intended to work less than 40 hours per week. The highest proportion of students (29%) who preferred part-time were those who opted for family medicine (not shown in table). Looking at those who preferred family medicine, 80% of the women and 68% of the men intended to work part-time.

### Motivational factors

Men and women differed somewhat in the importance they attached to the seven motivational factors (see Table 3). Women rated *interesting content*, *a lot of patient contact* and *combining work with family* significantly higher than men. Still, when the motivational factors were listed from highest to lowest rating, men and women had an almost identical ranking order. The only difference in rank was that *patient contact* was rated higher than *technical skills* among women, whereas the opposite was the case for the men.

**Table 3:** Rating (Likert scale 1–5) of motivational factors for specialty preference

**Motivational factors**	**Women**	**Men**	**p**
	**N = 215**	**N = 157**	
	**Mean (SD)**	**Mean (SD)**	
Interesting content	4.7 (0.5)	4.5 (0.6)	**.004**
A lot of direct patient contact	4.1 (0.9)	3.9 (0.9)	**.013**
In line with technical skills	4.1 (1.1)	4.0 (1.0)	NS
Combining work with family	3.9 (1.1)	3.6 (1.1)	**.020**
Career prospects	3.3 (1.0)	3.4 (1.0)	NS
Good salary	3.1 (0.9)	3.2 (1.1)	NS
Research opportunities	2.8 (1.2)	2.9 (1.2)	NS

*Note:* Significant gender differences were measured using a non-parametric test (Mann–Whitney *U* test); significance level was set at p < .05. NS = not significant. p < .05 in bold.

A total of 120 participants added a motivational factor of their own. About a third of these described a good working climate and having nice colleagues as important when choosing specialty (not shown in table). Other motives were: having possibilities to develop, being able to choose where to live and having variation in duties. The possibility to choose where to live was primarily mentioned by those preferring family medicine.

### Motivational factors associated with specialty preference

Both gender similarities and differences were disclosed in the associations between motivational factors and specialty preference (see Table 4). For both women and men, a high rating for *technical skills* and a low rating for *combining work with family* were associated with a surgical preference. It was only among women that *good salary* was positively associated with preferring surgical specialties. Women and men who opted for non-surgical specialties such as internal medicine or pediatrics generally rated *research opportunities* high. Among women, a high rating of *interesting content* was linked to a non-surgical specialty. For both men and women, valuing *patient contact* had a positive association with a preference for family medicine. Women who preferred family medicine generally rated *combining work with family* and *good salary* high and rated *interesting content*, *technical skills* and *career prospects* low. Male and female students who were undecided generally valued being able to combine work with family. Women who were uncertain generally rated *patient contact* low.

**Table 4:** Motivational factors associated with specialty preference

		**Women**		**Men**	
		**N = 214**		**N = 154**	
		**OR**	**p**	**OR**	**p**
**Surgical specialties**	Combining work with family	0.4 (0.3–0.6)	**.000**	0.4 (0.3–0.7)	**.000**
	Good salary^a^	1.9 (1.2–3.0)	**.004**	0.8 (0.5–1.3)	NS
	In line with technical skills	1.4 (1.0–1.9)	**.034**	1.9 (1.2–3.0)	**.011**
	Lots of direct patient contact	0.9 (0.6–1.3)	NS	0.6 (0.4–0.9)	**.018**
**Non-surgical specialties**	Research opportunities	1.4 (1.0–1.9)	**.023**	1.9 (1.3–2.9)	**.001**
	Good salary	0.4 (0.2–0.6)	**.000**	0.8 (0.5–1.3)	NS
	Interesting content	2.4 (1.0–5.5)	**.048**	1.7 (0.8–3.9)	NS
**Family medicine**	Lots of direct patient contact	3.7 (1.8–7.9)	**.001**	2.9 (1.4–6.0)	**.005**
	Career prospects^a^	0.4 (0.2–0.7)	**.002**	0.8 (0.4–1.5)	NS
	Combining work with family	2.3 (1.3–4.2)	**.004**	1.6 (0.9 –2.9)	NS
	Good salary	1.9 (1.1–3.3)	**.018**	1.7 (0.9–3.2)	NS
	Interesting content	0.4 (0.2–0.9)	**.024**	0.8 (0.4–1.9)	NS
	In line with technical skills	0.6 (0.4–1.0)	**.045**	0.7 (0.5–1.2)	NS
	Research opportunities	0.8 (0.6–1.3)	NS	0.6 (0.4–1.0)	**.031**
**Uncertain**	Combining work with family	2.1 (1.5–3.1)	**.000**	1.8 (1.2–2.7)	**.005**
	Lots of direct patient contact	0.6 (0.4–0.8)	**.003**	1.0 (0.7–1.5)	NS

*Note:* Specialty preference (outcome) = modeling the probability of choosing it (not choosing it = ref.). Mediators = motivational factors (probability of choosing a specialty preference). OR = odds ratio (95% CI = confidence interval). Significance was set at p < .05. NS = not significant. p < .05 in bold. ^a^Significant interaction term with gender in separate analyses on each motivational factor.

When gender as an interaction term was studied in the association between motivational factors and specialty preferences, two significant differences were found. First, a high rating of *good salary* had a stronger positive association with the women’s preference for surgery (OR 1.9) than it had for the men’s (OR 0.8). Second, women’s preference for family medicine had a stronger negative affiliation with *career prospects* (OR 0.4) than it had for men (OR 0.8).

The linear regression analysis investigated any association between ideal working hours and combining work with family (not in table) and showed that for women – not for men – the number of working hours correlated with how they rated *combining work with family* (for women p = .000 and for men p = .389). Opting for part-time meant a high rating of *combining work with family* and conversely: a low rating meant full-time or more.

## Discussion

This study aimed to investigate and compare male and female students’ specialty preferences and the motives behind them. Our results showed almost no gender differences in the specialties the students opted for. Moreover, men and women had an almost identical ranking order of the motivational factors. Male and female students did differ in the motivational factors that were associated with their specialty preference. However, just two statistically significant gender differences were revealed when using gender as an interaction term. A majority of the women, compared with a good third of the men, intended to work part-time. It was only for women that the ideal number of working hours was linked to the importance they attached to having time for family.

### Gender and career preferences

In contrast to earlier studies on medical students, we did not find any significant difference between men and women in their preference for surgery [11,12,21]. Our results were supported by a study conducted in all Swedish medical schools where no differences in specialty preference between male and female last-term medical students were revealed [30]. Also, earlier research has shown a trend where the proportions of female medical students who prefer male-dominated specialties are increasing [19,21]. Still, this equal distribution between men and women clashes with the horizontal gender segregation in medical specialties seen in Sweden and other Western societies [7,9]. This could either mean that women will continue to increase in male-dominated specialties or that the problem lies after graduation. In a Norwegian study, female and male residents were equally distributed when starting their first specialty training, but fewer women than men finished their specialty training in surgery [31]. Hence, there seem to be barriers such as masculine homosociality, with men preferring men (and excluding women), lack of social support and a scarcity of role models that might only become evident during residency.

That more women than men opted for gynecology and pediatrics could be explained by female physicians being numerous in these specialties over the past decades and hence there are more same sex role models. Also, the students were on gynecological and pediatric training at the time of the questionnaire.

Both male and female students seemed to value patient contact and combining work with family more than career prospects and good salary. This could be explained in part by the fact that a majority of the students lived with a partner and by the age of twenty-seven, family-life might seem close in time. Our results are in concert with another Swedish study, where male and female physicians mainly showed similarities in their specialty motives; interesting content, patient contact and a good working environment were important for both women and men [32]. Yet, our results contrast with previous studies from other countries where mainly women considered work and time-related aspects and patient orientation whereas men considered technical challenge, salary, career prospects and prestige when choosing specialty [11,15,16,18]. Both Norwegian and Swedish students [19,30] compared with students from, for example, the Netherlands and the U.S. distribute themselves more equally in their specialty preferences and also in how they rate the motives for them [10,12]. Perhaps this could be explained by different national contexts. The fact that both Sweden and Norway have more far-reaching gender equality legislation compared with countries like the U.S. and the Netherlands [26], is probably reflected in the values among the medical students. Thus, strong norms of gender equality as a frame of reference might affect the students’ career preferences and their motives for them.

In a study on Swedish residents, men and women in male-dominated specialties (such as surgery) attached similar importance to combining work with family, whereas those in specialties with more women differed in their priorities [32]. The same pattern can be seen in our results as well: men and women who opted for surgical specialties had the same low rating in combining work with family, whereas among those who preferred family medicine it was only women – not men – who considered time for family to be important. Those who opted for family medicine also chose part-time to a higher degree. It seems that women consider family medicine a family friendly specialty. This was consistent with women’s part-time preference being linked to having time for family. Thus, for women family medicine and part-time practice seem to be a strategy to combine work with family duties. For men, the choice of part-time and family medicine is about something else, which remains unmeasured.

Being uncertain and preferring non-surgical specialties were associated with relatively few motivational factors. This could be because these two groups were the most diverse. In the uncertain group there seemed to be a group of female students who were not motivated by patient contact and a group of male and female students who wanted combine work with family. Perhaps this means that if you want to be able to combine work with family in a satisfying way, career choices are much harder to make. Also, it is interesting that female students who went against a traditional gender pattern – being female and less concerned with patient contact – seemed to find it harder to choose a specialty.

A considerable number of students added a good working environment and nice colleagues when asked if something other than the stated motivational factors would affect their specialty preference. This finding is supported by a Swedish study where male and female students described how their reception among colleagues was important when making their career choices [33]. Also, an American study found that the reason for more men choosing surgery could not be explained by the women being deterred from surgery during their clinical rotations but rather that they received more support elsewhere [1].

To sum up, our results suggest that gender segregation is not just a matter of individual choices and gender-dichotomized preference; instead we found that contexts structure choices. First, in contrast to several other Western societies, Swedish men and women have very similar specialty preferences at the time of their graduation. Second, in the male- dominated surgical specialties both male and female students prioritize family low, whereas for women work-family balance is a major motivational factor to choose family medicine. Third, the importance of social support at the workplace was reflected in the students’ addition of a good working climate as an important factor for specialty preference.

### Part-time ideal

Almost half of the medical students who were standing on the doorstep of working-life preferred a part-time practice. In Sweden, 29% of all female physicians work part-time, which can be compared with a majority of the female students in our study who planned for part-time practice. In a similar vein, 17% of the male physicians work part-time, compared with a good third of the male students who intended to do so [34]. If these students’ preferences stay on and they get their way, the proportion of part-time practicing physicians could be almost doubled within a few decades. In Sweden, parents with small children and a full-time job have a legal right to work part-time (75%). Perhaps, medical students believe that this, together with an expected future shortage of doctors mean that they will be able to negotiate their working hours. In a Nordic report on the future supply of physicians, 20% of all Swedish physicians were expected to practice part- time in 2020 [35]. The report made an underestimation, as 23% of all Swedish physicians practiced part-time already in 2009 [34]. This raises a concern. If a new generation of doctors successfully negotiates part-time practice the shortage of doctors will be even larger than expected. There is however studies indicating that these students will not get what they wish for. Potential medical students in the U.S. also expected medicine to offer the possibility of part-time [1]. This is surprising, as historically the medical profession has been known for its long hours. In most specialties part-time is not a possibility. Structural and cultural barriers are important obstacles for part-time work. Despite a new generation of physicians who value work-life balance [22] the American trend over the last few decades moves towards working long weeks, mainly because of structural constraints [1]. American physicians who work part-time report less pay for the same work-related expectations and long-term sacrifices such as not being promoted and also criticism from colleagues [1]. Negative attitudes in the workplace toward part-time physicians have been described in Scandinavian studies as well [32,36]. As the students in this study seem to value the support from colleagues, this suspiciousness toward part-timers will be a rude awakening for the Swedish students when they enter the medical profession.

When the students were asked directly how important it was to be able to combine work with family, both men and women rated it higher than career possibilities. However, when using regression analysis to study the association between combining work with family and ideal work-time it was clear that it was mainly the female students who planned for part-time in order to have time for family responsibilities. This also means that the female students realize that more working hours mean less time for the family. Earlier research showed that female medical students and physicians were more ready than their male peers to compromise career aspirations for family life [17,22,32,33]. Thus, even if Sweden has relatively strong parental leave and childcare provisions, there are still differences in how male and female future physicians plan their careers. In concert with our results, it has been shown before how Swedish medical students receive gendered advice; men were encouraged to stick to what they aspired for and to let family interests come second whereas women were advised to choose a family-friendly specialty [33]. In sum, even if male and female students have similar specialty preferences, it was mainly women and especially those opting for family medicine that planned for work-family balance. However, this did not seem to affect their specialty preference, as there were no gender differences in opting for family medicine and surgical specialties.

### Strengths and limitations

The study population covered six classes of medical students and the response rate was high (89%). Seven out of ten motivational factors were useful when measuring their link to specialty preference. Some limitations in the current study warrant a discussion. This was a cross-sectional study and therefore one should be cautious about causality between motivational factors and specialty preference. Probably, they were both influenced by a general attitude toward work and the social discourses associated with the study being conducted at a Swedish university. The results were derived from a single medical school, which means that our findings were not necessarily representative of medical students in general. A majority of the students have however moved to Umea from all parts of Sweden. The participants were last-year students and still had to complete an internship that lasts at least one and a half year before choosing a specialty, which means they had quite some time to change their mind. This means that students’ preferences during medical school can, at its best, be used as an indication of what specialty they finally end up in. Using fixed alternatives together with a fixed scale implies a risk of neglecting factors of importance for career choice. Looking at the motivational factors that the students added themselves, we realized that we probably missed some important motives such as working climate and the possibility to choose where to live.

## Conclusions

The gender similarities in the medical students’ specialty preferences should be seized by the health care system in order to counteract the horizontal gender segregation in the physician workforce of today. One must acknowledge that Swedish medical students do not have dichotomized motivations for their specialty preference based on gender; instead men and women mainly show similarities. There is also a need to discuss gender and the interface between work and family life in medical schools in order to open up for a more equal gender distribution of time spent at the hospital and at home doing unpaid work.

## Competing interests

The authors declare they have no competing interests.

## Authors’ contributions

All five authors have made contributions to the study design, acquisition and interpretation of data. TLJ, PV, KH and EEJ was responsible for making the questionnaire. SD performed the statistical analysis to which KH, EEJ and PV made important contributions. SD drafted the manuscript and EEJ and KH made substantial contribution in writing the manuscript. All authors have been involved in revising the manuscript critically for important intellectual content and have read and approved the final manuscript.

## Pre-publication history

The pre-publication history for this paper can be accessed here:

http://www.biomedcentral.com/1472-6920/13/39/prepub
